# How Social Media Use at Work Affects Improvement of Older People’s Willingness to Delay Retirement During Transfer From Demographic Bonus to Health Bonus: Causal Relationship Empirical Study

**DOI:** 10.2196/18264

**Published:** 2021-02-10

**Authors:** Yiming Ma, Changyong Liang, Dongxiao Gu, Shuping Zhao, Xuejie Yang, Xiaoyu Wang

**Affiliations:** 1 School of Management Hefei University of Technology Hefei China; 2 Key Laboratory of Process Optimization and Intelligent Decision-making of Ministry of Education Hefei China; 3 The First Affiliated Hospital, Anhui University of Traditional Chinese Medicine Hefei China

**Keywords:** social media, older workers, social support, work ability, delayed retirement

## Abstract

**Background:**

With the increased older population in China and the subsequent reduced labor force, the “demographic bonus” is disappearing. The Chinese government proposed a Healthy China strategy in 2017. The transfer of the demographic bonus to a “health bonus” extended the working life of people and reduced the negative impact of the population’s aging on the labor force structure.

**Objective:**

This research focuses on the effect of older workers’ social media usage at work on their work ability (related to both physical and mental health) and thus their willingness to delay retirement.

**Methods:**

The questionnaire respondents were older than 55 years, and they obtained the questionnaire from social media, from June to July 2018. A total of 1020 valid questionnaires were collected, and SmartPLS 3.28 (SmartPLS GmbH) was used to analyze the data. Effects were analyzed using 2-tailed *t* tests.

**Results:**

(1) Use of social media at work can improve information support (*t*_14_=13.318, *P*<.001), emotional support (*t*_14_=13.184, *P*<.001), and self-efficacy (*t*_14_=6.364, *P*<.001) for older people; (2) information support is the main factor affecting the self-efficacy of older workers (*t*_14_=23.304, *P*<.001), as compared with emotional support (*t*_14_=1.799, *P*=0.07); (3) the impacts of emotional support on work ability (*t*_14_=8.876, *P*<.001) and work stress (*t*_14_=9.545, *P*<.001) are generally higher than those of information support (*t*_14_=4.394, *P*<.001; *t*_14_=5.002, *P*<.001); (4) self-efficacy has an impact on work ability (*t*_14_=5.658, *P*<.001) and work stress (*t*_14_=4.717, *P*<.001); and (5) the impacts of work ability (*t*_14_=8.586, *P*<.001) and work stress (*t*_14_=8.579, *P*<.001) on retirement willingness are greater than those of emotional support (*t*_14_=2.112, *P*=.04) and information support (*t*_14_=4.314, *P*<.001).

**Conclusions:**

Our study confirms that the use of social media at work has a positive impact on older workers. Based on the findings, we have put forward proposals to extend people’s working lives and help governments implement health bonus policies. In the future, we will compare the different values of willingness to delay retirement among older people in different occupations and different cultures.

## Introduction

### Background

The problems associated with an aging population have become a worldwide challenge, affecting multiple countries and regions. According to a World Health Organization report from late 2017, the proportion of the world’s population older than 60 years will double by 2050, increasing from 11% to 22%. According to estimates, by 2050, the absolute number of people older than 60 years will increase from 605 million to 2 billion [1]. This aging population means that the “demographic bonus” has disappeared in a large number of countries. The demographic bonus refers to the situation where a falling birth rate reduces the number of minors, reduces the burden on the family, and forms a relatively rich labor force, which is conducive to economic development [2]. However, the aging population also means that the labor force’s share of the population is falling, the dependency ratio is rising (ie, the dependency of older people and children), and the economy is developing slowly because of the shortage of labor. Therefore, it is necessary to find new sources of labor to reduce the negative impact of a labor shortage.

While high-income countries have the challenges of an aging population and declining demographic bonus, they also have a population of older adults (older than 60 years) who are healthier [3], with better working capabilities, compared with those in low-income countries. Therefore, developing human resources among older adults and promoting the generation of a second round of a demographic bonus has become a problem that needs to be solved by all countries troubled by the challenges of an aging population [4]. The Chinese government proposed a Healthy China strategy in 2017, promoting the transformation of the demographic bonus to a “health bonus,” and improving working life, which will reduce the aging population’s negative impact on the structure of the labor force. The health bonus refers to the situation where labor productivity is improved to alleviate the labor shortage caused by the aging population; this provides a foundation for the healthy growth of the population of older adults who have delayed retirement, as well as adults of childbearing age, by improving the health of the workforce [5].

Determining how to maintain and improve the physical and mental health level of older workers and optimize the working ability and work experience of older adults has become an important prerequisite to realize the health bonus. Compared with other age groups, older individuals are more susceptible to psychological and mental problems [6]. Effective social support can prevent and alleviate psychological problems and improve the physiological health of older adults, to a certain extent [7]. Therefore, increasing older adults’ access to social support is of great significance to extending their working life and delaying their retirement. Especially for older working adults, different types of social support can not only help older workers to improve their physical and mental health but also help them to acquire work-related knowledge and skills and prevent the decline in work that is often caused by aging.

With the development of information and communications technology and the use of social media, access to social support is no longer realized through a single offline channel but can be realized through interactive online and offline channels [7]. Thus, using social media can help older people obtain social support. At present, social media has become an integral part of work. Some people think that the use of social media at work can have negative effects (such as reducing productivity and increasing disturbances) [8]. However, recent studies have shown that social media plays a positive role in work. By using social media at work, people can quickly assign work tasks and report work status and consultation, and workers can obtain different types of social support, which can have a positive impact on people in many ways. This social support can establish and strengthen connections among colleagues, help workers collect professional information, and promote knowledge and resource sharing [9]. Moreover, these behaviors can also promote people’s achievement of self-efficacy and improve their self-confidence at work [10]. In addition, non–work-related social media use behaviors (such as entertainment behaviors on social media) can reduce work stress and psychological problems [9]. And interactions on social media related to health information (such as competitive step counting) can also promote exercise around the workplace and maintain people’s level of health [11]. In conclusion, the existing research has suggested that the use of social media at work can improve workers’ mental health and work ability.

However, the existing research on social media mainly targets “ordinary” employees, resulting in a gap in research on older workers (older than 60 years). Research targeting older workers focuses on their working status, with a gap in research on their willingness to delay retirement. For these older workers, the main factors affecting their retirement are not only economic factors but also health level and job satisfaction [12,13]. The use of social media at work can improve the physical and mental health of older workers to a certain extent and improve their working ability. Furthermore, it can improve the performance of older workers and make them more likely to achieve job satisfaction. Thus, the use of social media at work can affect older workers’ willingness to delay retirement. Currently, there is a lack of empirical evidence to confirm that the use of social media at work can affect delayed retirement. In addition, further understanding the demands of employees older than 60 years, as well as how to enhance their ability to work and increase their willingness to delay retirement, promotes the development of this human resource and is of great economic and social value and important theoretical significance. Therefore, this paper puts forward the following questions to explore the impact of social media use at work on the intention of older workers to delay retirement:

1. How does social media use at work improve older workers’ physical and mental health, and how does it affect work ability and job burnout?
2. Will social media use at work affect the expected working duration of older workers in the future?

Based on theories relating social support and work ability, this paper studies the work ability and working duration of older adults from the perspective of social media. This paper narrows the focus from all sectors of the community to the sector of older workers, so as to actively improve their workplace conditions and create a better environment for them, thus promoting the health bonus.

### Literature Review and Hypotheses

#### Social Media at Work

Social media is a highly interactive platform based on information and communication technology. Individuals and communities can share, create, discuss, and modify user-generated content through these platforms such as WeChat, Facebook, Twitter, and QQ [14]. The functions of social media include identity, conversations, sharing, presence, relationships, reputation, and groups [14]. The use of social media at work has become a common phenomenon [15]. Some scholars believe that the use of social media at work will distract employees’ attention and lead to a decline in work efficiency [8]. However, from a social support perspective, social media used at work is beneficial [16].

Social support is defined as the assistance an individual can access from the social resources of his or her social network [17]. Social support can be divided into four categories: material, emotional, information, and companion support [17]. The social support obtained at work mainly includes information support and emotional support [18]. These different types of social support are important for people to access work-related resources and improve their work-related abilities. Through the use of social media at work, people can more easily access various types of social support. Therefore, we believe that the positive effects of social media use at work include the following aspects:

First, the use of social media at work can increase social support for employees [18]. This kind of social support includes emotional and information support. Social media can make it easier for employees to connect and interact with colleagues [18]. This kind of interaction can facilitate the sharing of work experiences and exchanges of knowledge among employees (information support) [19]. For older people, the use of social media can help them maintain their existing relationships, while the process of learning the method to use social media is also a process of acquiring social support (information and emotional support) [20]. In addition, older people tend to need more attention and support [21]; for older workers, this kind of attention and support is extraordinarily important (emotional support). The characteristics of social media, such as conversations, sharing, presence, and relationships, can increase the acquisition of emotional support [20]. Based on the literature cited above, we believe that the use of social media at work can improve the level of social support for older employees, including emotional support and information support. Therefore, we propose the following hypotheses:

H1: The use of social media at work can improve emotional support for older workers.

H2: The use of social media at work can improve information support for older workers.

Second, social support gained through the use of social media can also increase self-efficacy. Self-efficacy refers to an individual’s belief in his or her capacity to execute behaviors necessary to achieve specific performance outcomes [22]. At work, the level of self-efficacy often determines the individual’s work ability and work performance. Factors that affect self-efficacy include knowledge, skills, experience, and social support [22]. Social support can significantly regulate an individual’s self-efficacy [23]. On the one hand, social support can promote the acquisition of new knowledge and skills (information support), thereby improving self-efficacy [23]. On the other hand, emotional support can also affect people’s self-efficacy. Self-efficacy is constantly changing on a daily basis [24]. The change in self-efficacy is often due to changes in interpersonal relationships [25]. As an important tool for maintaining an individual’s interpersonal relationships, social media can help people maintain their social networks in a working environment and help them give or get emotional support. In addition, for older people, the use of social media directly affects an individual’s sense of self-efficacy. Involvement in and use of social media can heighten an individual’s sense of general self-efficacy, which will increase with a deepening use of social media [26]. In summary, the use of social media can improve people’s self-efficacy at work in different ways. Based on the above conclusions, we put forward the following hypotheses:

H3: The use of social media at work can improve older workers’ self-efficacy.

H4: The emotional support accessed from social media can improve older workers’ self-efficacy.

H5: The information support accessed from social media can improve older workers’ self-efficacy.

#### Social Support and Work Ability

Work ability is defined as the sum of the factors enabling an employed person in a certain situation to manage his or her working demands successfully [27]. Older workers have abundant work experience and good working skills, but they inevitably experience a decline in physical fitness and cognitive ability with an increase in age. Therefore, for older people, work ability refers to the physical and mental health level that can meet the needs of the work [28].

Effective social support is of great significance to the work ability of employees, especially for older workers. Effective emotional support is an important way for people to maintain and improve their working ability. For example, Pohl and Galletta have demonstrated that emotional support provided by a supervisor in the workplace can improve employees’ work ability and reduce their sense of fatigue [29]. A study by Karlsson et al showed that emotional support can help individuals better cope with injuries and combat the decline in work ability caused by injuries [30]. In addition, the work ability level is often associated with the mental health level. Especially for older workers, mental illness is an important reason for a decline in work ability [31]. Emotional support can help individuals prevent and alleviate the harm brought by mental illness [17].

Similarly, information support can also have an important impact on an individual’s work ability. Information support at work mainly includes information sharing and the exchange of new knowledge and skills [19]. For older workers, learning new knowledge and skills can improve their work efficiency and prevent the decline in work ability caused by aging [32]. In addition, non–work-related information sharing also can improve older people’s ability to work. For example, Edmunds et al confirmed that the support of colleagues related to health information can promote workplace exercise, improve workers’ health level, and enhance their work ability [11]. Workplace exercise can not only improve the physical health of older adults but also improve their mental health, which is of great significance for maintaining their work ability [33]. To sum up, we believe that both emotional support and information support will impact the working ability of older workers. Therefore, we hypothesize the following:

H6: Emotional support can improve older workers’ work ability.

H7: Information support can improve older workers’ work ability.

Work stress reflects an interaction between individual characteristics and an individual’s response to work characteristics [34]. Ganster and Rosen argued that work stress is a process where workers experience mental and physical changes in the short or long term caused by mental experiences and demands at the workplace (the source of stress) [35]. Work stress has negative effects on individuals, causing such physical problems as headache, heart disease, elevated blood pressure, gastropathy, and insomnia, and psychological disorders including depression, hostility, and withdrawal [36].

Good social support can improve employee performance and ease work stress [37]. Emotional support can help individuals better cope with work stress. For example, research by Yang et al shows that peer support and subjective emotional support can effectively help older employees cope with work stress [38]. Moeller and Chung-Yan also confirmed that emotional support from supervisors can improve employees’ mental health and reduce their work stress [39]. Similarly, information support from supervisors can also effectively help employees cope with work stress [39]. This is because credible workplace information can reduce an employee’s sense of unpredictability and powerlessness, which reduces psychological distress and work stress [39]. In addition, research by Chrisopoulos et al suggests that although emotional support can improve people’s ability to cope with work stress, it cannot change the objective stress situation; information support related to tasks or technologies can improve people’s work efficiency and reduce their work stress [40]. Based on the above conclusions, we put forward the following hypotheses:

H8: Emotional support can relieve older workers’ work stress.

H9: Information support can relieve older workers’ work stress.

#### Self-Efficacy and Work Ability

Self-efficacy greatly influences an individual’s abilities and work stress [41]. First, individuals with higher self-efficacy can cope with work stress more effectively and are more positive about their work. Skaalvik and Skaalvik found a negative correlation between teacher stress and teacher self-efficacy [42]. Research by Lloyd et al shows that self-efficacy can improve people’s intrinsic work motivation and reduce work stress [43]. Self-efficacy can also improve work ability in different dimensions. Self-efficacy can influence people’s work ability by improving an individual’s health, self-confidence, social function, and other factors [44]. Improving work-related self-efficacy and self-management can improve employees’ work ability [45]. Higher self-efficacy can also reduce the incidence of mental illness [46]. A healthy state of mind can have a positive effect on work ability and status [31]. Based on the above findings, we believe that self-efficacy can improve older people’s work ability and relieve their work stress. Therefore, we put forward the following hypotheses:

H10: Self-efficacy can improve older workers’ work ability.

H11: Self-efficacy can alleviate older workers’ work stress.

#### Willingness to Delay Retirement

The main influencing factors on the duration of older individuals’ work lives are their individual health and financial pressures [12]. An individual’s subjective will also plays a decisive role [12]. For example, Skaalvik and Skaalvik found that work stress is an important factor affecting one’s willingness to retire [42]. Heavier work stress can lead to early retirement, while lighter work stress can prompt individuals to delay their retirement, even motivating teachers older than 65 years to continue working. The individual’s sense of satisfaction about nonmaterial factors, such as enjoying working and social recognition, will affect the willingness of adults who reach retirement age to delay retirement [47]. Highly competent (as far as work ability) individuals tend to have greater enthusiasm for work. For individuals with greater enthusiasm for work, retirement can lead to a huge psychological gap [48]. In addition, when work is challenging, people with high work ability tend to be more willing to work longer and delay their retirement [49]. Therefore, for individuals with a passion for work and higher level of work ability, extending the duration of their working life as much as possible can help them maintain their existing social status and enjoy the fun of work. Therefore, we put forward the following hypotheses:

H12: Work stress will have a negative impact on older workers’ willingness to delay retirement.

H13: Work ability will have a positive impact on older workers’ willingness to delay retirement.

Finally, the social support gained at work also influences an individual’s willingness to retire. According to Hofstetter and Cohen, for older adults, emotional support from colleagues (such as kindness and acceptance) will enhance people’s happiness and affect their retirement intention [50]. Emotional support provided by an organization (such as caring for workers’ general health) will affect the sense of belonging of older workers and their intention to retire [50]. In addition, continuous learning and development (information support) opportunities provided by organizations relate to the preference for postponing retirement [51]. By contrast, insufficient professional information support by colleagues will lead to career and job stagnation, which may lead to premature retirement [50]. Therefore, the work duration of older individuals is affected by social support. In summary, we believe that work stress, work ability, and social support impact older individuals’ willingness to delay retirement. Therefore, we put forward the following hypotheses:

H14: Information support will have a positive impact on older workers’ willingness to delay retirement.

H15: Emotional support will have a positive impact on older workers’ willingness to delay retirement.

**Figure 1:**
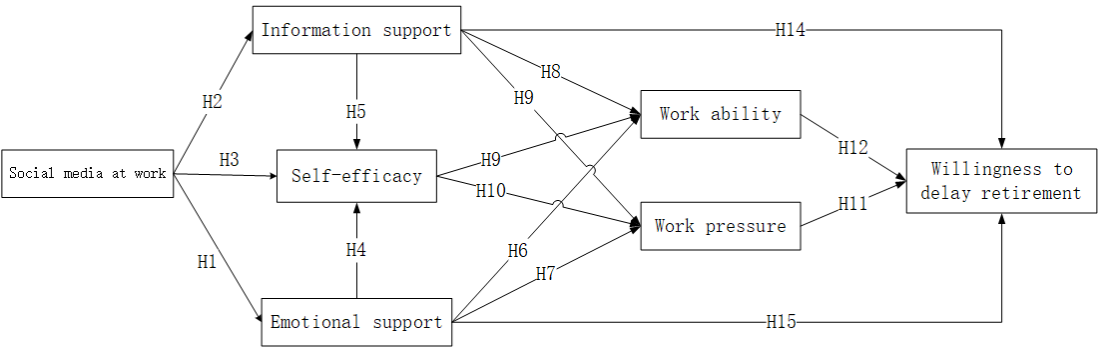
The research model.

In summary, this study’s proposed model studies the willingness of older workers to delay retirement from the perspective of their social media use at work, examining numerous hypotheses as depicted in Figure 1.

## Methods

### Scale Development

In this study, the main measurement variables included social media at work, social support (emotional and information), work ability, willingness to delay retirement, work stress, and self-efficacy. The questionnaire adopted the form of a Likert 7-point scale. An answer from 1 to 7 indicated the degree to which respondents agreed with the question; 1 meant completely disagree, and 7 meant completely agree. All constructs’ measures used in this study are listed in Multimedia Appendix 1 [9,52-59].

A scale showing the effects of social media at work was compiled on the basis of previous studies on an effective scale and was amended to adapt to this study’s background. The questions to measure included those about frequency, improving work efficiency, improving communication skills, relieving pressure, learning on the job, and undertaking recreational activities [9,52,53]. In the presurvey sample, the scale had good internal consistency reliability, with a Cronbach α of .902.

The scale of work ability and willingness to delay retirement was based on the work ability index [54]. The Chinese version of the scale shows good reliability and validity [55]. We included 7 items and adapted them to fit the current context. The measured items for work ability included the self-evaluation of work ability, mental adaptability to the current work situation, and physical adaptability to the current work situation, as well as a work performance evaluation. The measured items for willingness to delay retirement included the willingness to retire, self-predicting of future work ability, and willingness to delay retirement. In the presurvey sample, the scale had good internal consistency reliability, with a Cronbach α of .873.

To measure social support, we adopted the social support scale by Cohen and Wills [60]. The scale has shown good reliability and validity in previous studies [56,57]. We adapted the original scale to our needs; the measure of dimensionality included information support and emotional support. In the presurvey sample, the scale had good internal consistency reliability, with a Cronbach α of .901.

Work stress was based on a job content questionnaire [61]. The Chinese version of the scale shows good reliability and validity [62]. The scale included 4 items that refer to quantitative, demanding aspects of the job (eg, time pressure, working hard, excessive work). In the presurvey sample, the scale had good internal consistency reliability, with a Cronbach α of .796.

The earliest general self-efficacy scale was compiled by Schwarzerin in 1981, and the Chinese version was compiled and used in 1995 [58]. The scale has shown good reliability and validity in previous studies [58]. We adapted the original scale to our needs and included 4 items in the questionnaire. In the presurvey sample, the scale had good internal consistency reliability, with a Cronbach α of .789.

### Data Collection

The data collection process was divided into several stages. To ensure the quality of the questionnaire, 50 presurvey copies were randomly distributed in Bengbu, China; of these, 47 valid questionnaires were recovered. Some participants were interviewed to determine whether there were problems with the questionnaire, such as unclear language expression or rhetorical errors. Based on the presurvey results, we modified the questionnaire.

The formal questionnaire respondents (staff older than 55 years) were obtained through the Bengbu social security office, and they obtained the questionnaire from social media from June to July 2018. A total of 1500 questionnaires were issued, and 1291 were returned, giving a return rate of 86.1%. After eliminating invalid questionnaires (those that had many blank answers and a high repetition rate of answers), 1020 valid questionnaires remained, giving an effective rate of 79.0%. Table 1 shows the statistics for the demographics.

**Table 1:** The demographics of the sample.

Category		n (%)
**Sex**		
	Male	521 (51.1)
	Female	499 (48.9)
**Age (years)**		
	55-60	146 (14.3)
	61-65	629 (61.7)
	66-70	175 (17.2)
	>70	70 (6.9)
**Marital status**		
	Unmarried	61 (5.9)
	Married	772 (75.7)
	Divorced	76 (7.5)
	Widowed	111 (10.9)
**Educational background**		
	Elementary school	328 (32.2)
	Middle school	317 (31.1)
	High school	106 (10.4)
	College	122 (12.0)
	Master’s degree	147 (14.4)
**Income (**¥**)**		
	<1000	180 (17.6)
	1000-2000	216 (21.2)
	2000-3000	210 (20.6)
	3000-4000	216 (21.2)
	4000-5000	81 (7.9)
	5000-6000	64 (6.3)
	＞6000	53 (5.2)

## Results

### Model Overview

We used a structural partial least squares structural equation modeling (PLS-SEM) method to analyze the data obtained. The model framework was analyzed using SmartPLS 3.28 (SmartPLS GmbH). The PLS method has relatively loose requirements for the normal distribution of the research sample data and has flexibility in dealing with missing data. Therefore, PLS is suitable for exploratory factor analysis. In addition, PLS-SEM is a comprehensive method that can simultaneously examine all the relationships between the constructs in the measurement and the structural models and can also handle complex models with direct and indirect relationships [63,64]. Therefore, when the model complexity is high, PLS-SEM has more advantages than other methods.

### Measurement Model

The measurement model includes the following steps: First, SPSS 22 (IBM Corporation) was used for data analysis. The Cronbach α coefficient value was .880, greater than .80, indicating that the reliability of the questionnaire was good. The test results show a Kaiser-Meyer-Olkin value of 0.899 and a significance level of *P*<.001. These values indicate that the scale used in this paper has good structural validity, and that the questionnaire is suitable for factor analysis.

To avoid multicollinearity, we tested the data. The maximum variance expansion coefficient was 2.015, much lower than the prescriptive diagnosis of 5 [65]. Moreover, for the goodness of fit, the standardized root mean square residual was measured. The standardized root mean square residual has already been used as the goodness of fit method in PLS-SEM measurement [66]. Standardized root mean square residual values of less than 0.10 or 0.08 (in the more conservative version) are considered suitable [66]. In this study, the standardized root mean square residual was 0.045, less than 0.08. Thus, the model was very well adapted.

Table 2 shows the statistical data of factor loading, composite reliability, Cronbach α, and average variance extracted (AVE). According to Hair, the value of the Cronbach α coefficient should be above .7; in this study, the Cronbach α coefficient was between .792 and .880, indicating that the reliability of the questionnaire was good [65]. The composite reliability value ranged from 0.873 to 0.909, which was higher than 0.7, indicating that the questionnaire had good convergent validity [65]. In addition, the average variance extracted was greater than 0.5, indicating that the observed items explain the variance more than the error term [65] and that the model aggregation validity is relatively high.

**Table 2:** Construct reliability and convergent validity.

Construct items		Loading		CR^a^		Cronbach α		AVE^b^	
**WDR^c^**					0.878		.792		0.706
	WDR1		0.839		N/A^d^		N/A		N/A
	WDR2		0.818		N/A		N/A		N/A
	WDR3		0.863		N/A		N/A		N/A
**WS^e^**					0.891		.838		0.673
	WS1		0.798		N/A		N/A		N/A
	WS2		0.845		N/A		N/A		N/A
	WS3		0.811		N/A		N/A		N/A
	WS4		0.825		N/A		N/A		N/A
**ES^f^**					0.883		.823		0.653
	ES1		0.835		N/A		N/A		N/A
	ES2		0.821		N/A		N/A		N/A
	ES3		0.793		N/A		N/A		N/A
	ES4		0.783		N/A		N/A		N/A
**SMW^g^**					0.909		.880		0.625
	SMW1		0.792		N/A		N/A		N/A
	SMW2		0.777		N/A		N/A		N/A
	SMW3		0.822		N/A		N/A		N/A
	SMW4		0.759		N/A		N/A		N/A
	SMW5		0.789		N/A		N/A		N/A
	SMW6		0.804		N/A		N/A		N/A
**IS^h^**					0.875		.810		0.636
	IST1		0.807		N/A		N/A		N/A
	IST2		0.8		N/A		N/A		N/A
	IST3		0.8		N/A		N/A		N/A
	IST4		0.784		N/A		N/A		N/A
**SE^i^**					0.893		.840		0.676
	SE1		0.814		N/A		N/A		N/A
	SE2		0.812		N/A		N/A		N/A
	SE3		0.832		N/A		N/A		N/A
	SE4		0.831		N/A		N/A		N/A
**WAI^j^**					0.873		.806		0.632
	WAI1		0.78		N/A		N/A		N/A
	WAI2		0.802		N/A		N/A		N/A
	WAI3		0.8		N/A		N/A		N/A
	WAI4		0.799		N/A		N/A		N/A

^a^CR: composite reliability.

^b^AVE: average variance extracted.

^c^WDR: willingness to delay retirement.

^d^N/A: not applicable.

^e^WS: work stress.

^f^ES: emotional support.

^g^SMW: social media at work.

^h^IS: information support.

^i^SE: self-efficacy.

^j^WAI: work ability index.

Table 3 shows that the square root of each factor’s AVE value is greater than the other factor correlation coefficients, indicating that the questionnaire had good discriminant validity [65]. In summary, the model has good reliability and validity.

**Table 3:** Measurement model results.^a^

Constructs	WS^b^	WAI^c^	IS^d^	ES^e^	WDR^f^	SMW^g^	SE^h^
WS	0.820	—^j^	—	—	—	—	—
WAI	0.741	0.795	—	—	—	—	—
IS	0.495	0.510	0.798	—	—	—	—
ES	0.469	0.477	0.460	0.808	—	—	—
WDR	0.703	0.710	0.492	0.479	0.840	—	—
SMW	0.488	0.467	0.456	0.445	0.433	0.791	—
SE	0.481	0.516	0.726	0.412	0.475	0.485	0.822

^a^The numbers on the diagonal are the square roots of the variance shared between the constructs and their measures. Off-diagonal elements are correlations among constructs. For discriminant validity, diagonal elements should be larger than off-diagonal elements.

^b^WS: work stress.

^c^WAI: work ability index.

^d^IS: information support.

^e^ES: emotional support.

^f^WDR: willingness to delay retirement.

^g^SMW: social media at work.

^h^SE: self-efficacy.

^j^—: not applicable.

### Structural Model

**Figure 2:**
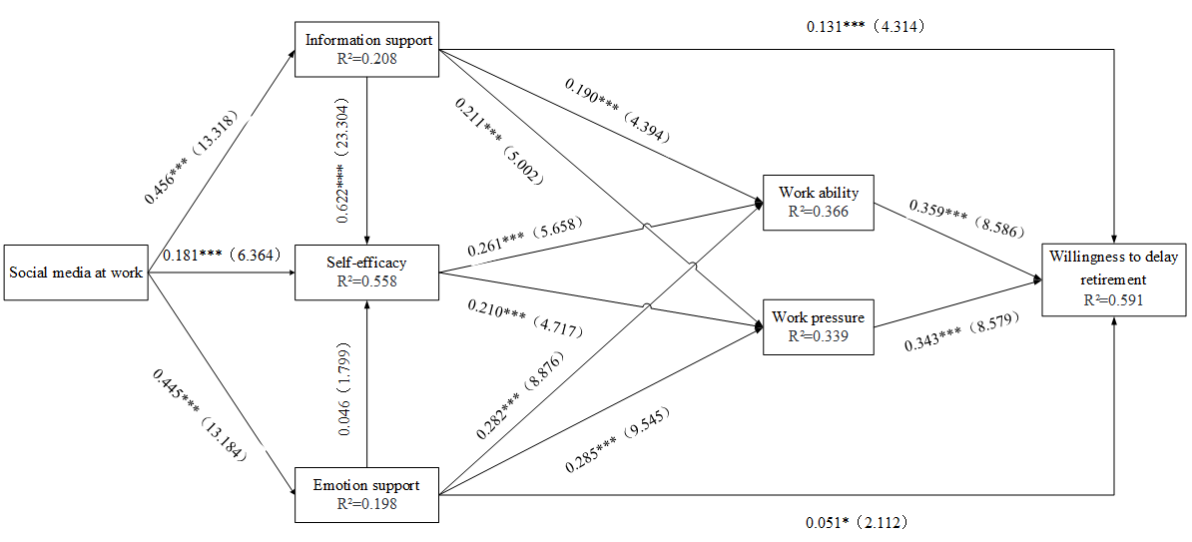
Model results. *P<.05, ***P<.001.

We used SmartPLS 3.28 to calculate the significance of the model path coefficients using bootstrapping with 3000 samples and 2-tailed *t* tests. The results of direct effects are shown in Figure 2 and Table 4. We also test the total and indirect effects of social media at work on willingness to delay retirement, and the results are shown in Multimedia Appendix 2.

**Table 4:** Structural parameter estimate.

Hypothesized path	SPC^a^	*t* value	*P* value	Results
H1: The use of social media at work can improve emotional support for older people.	0.445	13.184	<.001	Supported
H2: The use of social media at work can improve information support for older people.	0.456	13.318	<.001	Supported
H3: The use of social media at work can improve older people’s self-efficacy.	0.181	6.364	<.001	Supported
H4: The emotional support accessed from social media can improve older people’s self-efficacy.	0.046	1.799	.07	Not supported
H5: The information support accessed from social media can improve older people’s self-efficacy.	0.622	23.304	<.001	Supported
H6: Emotional support can improve older workers’ work ability.	0.282	8.876	<.001	Supported
H7: Information support can improve older workers’ work ability.	0.19	4.394	<.001	Supported
H8: Emotional support can relieve older workers’ work stress.	0.285	9.545	<.001	Supported
H9: Information support can relieve older workers’ work stress.	0.211	5.002	<.001	Supported
H10: Self-efficacy can improve older workers’ work ability.	0.261	5.658	<.001	Supported
H11: Self-efficacy can alleviate older workers’ work stress.	0.210	4.717	<.001	Supported
H12: Work stress will have a negative impact on older workers’ willingness to delay retirement.	0.343	8.579	<.001	Supported
H13: Work ability will have a positive impact on older workers’ willingness to delay retirement.	0.359	8.586	<.001	Supported
H14: Information support will have a positive impact on older workers’ willingness to delay retirement.	0.131	4.314	<.001	Supported
H15: Emotional support will have a positive impact on older workers’ willingness to delay retirement.	0.051	2.112	.04	Supported

^a^SPC: standardized path coefficient.

The hypotheses (H1, H2, and H3) that use of social media at work can improve social support for older people are supported. The effect levels of emotional support (standardized path coefficient=0.445, *t*_14_=13.184, *P*<.001) and of information support (standardized path coefficient=0.456, *t*_14_=13.318, *P*<.001), which are the two dimensions of social support, are quite similar. Compared with social support, social media has a slighter impact on self-efficacy (standardized path coefficient=0.181, *t*_14_=6.364, *P*<.001).

In the hypothesis (H5) of the relationship between social support and self-efficacy, the influence of information support on self-efficacy is confirmed (standardized path coefficient=0.622, *t*_14_=23.304, *P*<.001). However, the hypothesis (H4) on the effect of emotional support on self-efficacy proved to be untenable (standardized path coefficient=0.046, *t*_14_=1.799, *P*=0.07).

The hypotheses that social support has a positive effect on the work ability (H6, H7) and work stress (H8, H9) of older workers are confirmed. The influences of emotional support on the work stress of older adults (standardized path coefficient=0.285, *t*_14_=9.545, *P*<.001) and the ability to work (standardized path coefficient=0.282, *t*_14_=8.876, *P*<.001) are bigger overall than those of information support for older workers’ work ability (standardized path coefficient=0.190, *t*_14_=4.394, *P*<.001) and work stress (standardized path coefficient=0.211, *t*_14_=5.002, *P*<.001).

The hypotheses that self-efficacy has a positive effect on the working ability (H10; standardized path coefficient=0.261, *t*_14_=5.658, *P*<.001) and work stress (H11; standardized path coefficient=0.210, *t*_14_=4.717, *P*<.001) of older workers are confirmed.

The hypotheses that work stress (H12), work ability (H13), and social support (H14, H15) have an impact on the willingness of older workers to delay retirement are confirmed. Work stress (standardized path coefficient=0.343, *t*_14_=8.579, *P*<.001) and work ability (standardized path coefficient=0.359, *t*_14_=8.586, *P*<.001) have a greater impact on the retirement intention of older workers than does social support. The influence of emotional support (standardized path coefficient=0.051, *t*_14_=2.112, *P*=.04) on the intention to delay retirement, which is included in social support, is smaller than that of information support (standardized path coefficient=0.131, *t*_14_=4.314, *P*<.001).

Finally, we analyze the effect of social media use at work on older workers’ willingness to retire after adding control variables. The *t* test results show that sex, age, marital status, educational level, and income have no significant effect on older workers’ willingness to retire. This means that demographic characteristics have no significant effect on the analysis results.

## Discussion

### Findings

This paper examines the impact of social media use at work on elderly workers’ willingness to retire. The empirical results show that 14 of the 15 hypotheses in the research model are confirmed, and 1 hypothesis is not supported. Social media positively impacts elderly workers' willingness to delay retirement. Information support affects their self-efficacy more than emotional support does, while emotional support has a greater effect on work ability and work stress. However, self-efficacy also impacts work ability and the ability to regulate work stress. The impact of work ability and work stress on willingness to retire is greater than that of social support. More detailed results are given in Multimedia Appendix 2.

Our study confirms that the use of social media at work has a positive impact on older workers. The findings suggest that the use of social media at work can help older workers improve their social support and self-efficacy (H1, H2, and H3), which is very important for older people; effective social support can enhance their physical and mental health and reduce the occurrence of psychological disorders such as depression and anxiety [11,17,33]. Similarly, self-efficacy also has positive implications for the mental health of older people [44,46].

In a work environment, information support is the main factor affecting the self-efficacy of older workers, as compared with emotional support. This is a new finding. In previous studies, there was a significant correlation between social support and self-efficacy [23-25]. However, in this study, only information support impacts self-efficacy (H5), and emotional support has no influence on self-efficacy (H4). This result can be explained by the fact that for older workers, past work experience helps them build enough confidence to complete the work they are given. Therefore, emotional encouragement and support have no significant effect on their work self-efficacy. In addition, information support can help older people understand relevant information and learn new skills needed in their work. This is a major finding that reveals new ways to improve the self-efficacy of older workers.

The results also show that the impact of emotional support on work ability and work stress is generally higher than that of information support (H6-H9). This result can be explained by the fact that older workers have established practices for handling their work based on past experience, and the acquisition of new knowledge and new skills is only complementary to their own work ability. They are also experienced enough to cope with work stress. For older workers, who are moving toward the last years of their lives, effective emotional support such as a sense of achievement and satisfaction, as well as respect, can help them to work better and handle work stress. Therefore, the impact of information support on older workers is less than that of emotional support, in this context.

The results show that self-efficacy impacts work ability and ability to regulate work stress (H10 and H11). This result confirms the results of previous research [41]. We believe that this result is due to the specific life stage of older adults. The intellectual and physical decline caused by aging requires effective internal motivation to help older adults stay active at work and alleviate work stress. In addition, considering the results of H6 through H9, social support can alleviate work stress. We found that compared with self-efficacy, social support can more effectively alleviate work stress for older workers. Considering the sources of work stress, we believe that for older workers, when they are doing complex work, they are less able to regulate emotions internally, and external support is needed to help them moderate their emotions. This also confirms the research of Isaacowitz et al [67].

The impact of work ability and work stress on willingness to delay retirement is greater than that of social support. This result indicates that among the influencing factors for older workers’ willingness to delay retirement, the individual perceptions of work ability and work stress are the main factors. In addition, work ability and work stress have an equally important impact on one’s willingness to delay retirement, while social support has a relatively small impact on this willingness. This result suggests that older people’s willingness to delay retirement is mainly affected by work-related factors, and stronger work ability and lower work stress can extend their work duration and delay their retirement.

### Implications for Research

This study has several theoretical contributions.

First, our study links the use of social media at work to the willingness of older workers to delay retirement. This is a topic that has not been considered in the past. Past research on the use of social media at work was not specifically targeted at older workers, and there was limited research on their willingness to delay retirement. This study fills this gap. It also demonstrates the influencing mechanism of social media on the special group of older workers. This finding reveals the positive role played by the use of social media at work and its applicability across different groups.

In the course of the study, we explored the impact of social support, work ability, and work stress on older people’s willingness to delay retirement, which has never been explored before. The results show that an individual’s work ability (internal factor) and work stress (external factor) are mainly affected by external support, while internal subjective motivation (self-efficacy) plays a smaller role than external support. This finding can provide a theoretical basis for guiding the establishment of an effective incentive model for delayed retirement in the future.

Second, our study shows the special nature of older workers. The link between social support and self-efficacy has long been confirmed via past studies [23]. It is generally argued that all dimensions of social support have a positive impact on self-efficacy. However, in this study, the emotional support dimension of social support does not influence self-efficacy. This result suggests that in the working environment, the social support that older workers gain from using social media at work is more intentional; that is, the support is in getting help, gaining new knowledge, or reducing stress at work. This result confirms the results of previous studies [9].

At the same time, we also found that emotional support at work is more important than information support for older people in terms of work ability and work stress. This result shows the special nature of older workers. That is, as older workers are at a late stage of life, they need emotional support more than other dimensions of social support. This result also confirms that different dimensions of social support play different roles at different stages. This is an interesting finding that develops and extends social support–related theories.

Research has shown that self-efficacy is often an important factor to help individuals cope with work stress and improve work ability. This result also confirms the results of previous studies [42]. Combined with the impact of social support on self-efficacy (H4 and H5), the result shows that the self-efficacy of older workers often comes from their acquisition of technology experience and ability, while successful practices from their pasts weaken the role of external emotional incentives. Considering the differences between this study and previous studies (age differences, work and nonwork differences), the results of this study help to further understanding of the special nature of older workers.

### Implications for Practice

This study contributes to practice in the following ways:

Our study can provide a basis for relevant government departments in their development of a health bonus plan. Especially in China, the rapid aging and fertility decline of the overall population create an urgent need to develop a health bonus plan in line with national conditions. Moreover, our study can reduce resistance from older people when developing a deferred-retirement policy. The results of the study show that older people’s willingness to delay retirement is influenced by their work ability and work stress. Therefore, the differences between occupations and the actual situation of different types of groups of older adults can be taken into account during the formulation of a deferred-retirement plan, so as to make the plan more reasonable and effectively use the resources of the older adult population and promote the realization of the health bonus.

For companies, our study helps improve the management of, and optimize management plans for, older employees. Based on our conclusions, emotional support can better improve work ability and can reduce work stress more effectively for older workers. Therefore, companies can provide older employees with more emotional encouragement and care and can pay attention to their emotional state. From a social media perspective, companies should encourage and support older people in their use of social media at work, which is of positive significance. For example, company executives can show concern for older workers regarding their work status and mood by using social media. Additionally, colleagues can exchange work-related information, such as new knowledge and new job skills, or encourage each other with exercising by using social media.

The older workers’ acceptance and use of social media devices is important to realize positive aging; this is also an important prerequisite for realizing the health bonus. Our study confirms that using social media at work can improve both social support and self-efficacy, which can not only help older adults at work but also improve their mental health to prevent the physical and mental health problems caused by aging. This result can also help social media developers improve and develop social media features that are more suitable for older people.

### Limitations and Future Directions

Due to limited time and other constraints, our study has the following limitations:

First, we studied the impact of social media on older people’s willingness to retire. While this impact is confirmed, social support accessed from social media is only studied in terms of information support and emotional support. Social support has multiple dimensions, and our study does not explore the impact of these other dimensions of social support on older people; examples include objective social support and perceived social support dimensions, and the impact of the availability of support on future deferred-retirement intentions. Therefore, future studies will subdivide social support into these other dimensions and explore in detail the relationship between different social support dimensions, in order to understand the intrinsic relevance of social support to older people’s willingness to delay retirement.

Second, this study does not subdivide occupations or take into account the different characteristics of different occupations. Our study is incomplete, and in the future different occupations will be compared to explore the differences in the willingness to delay retirement among older people in different occupations.

Finally, the main object of study in this paper is a segment of the older population in Anhui Province, China. It is unclear whether the same results would be obtained in other provinces or countries due to cultural differences. Future research based on the results of this study will compare the willingness to delay retirement in different cultures.

### Conclusions

The need for deferred retirement has gained a general consensus in China and other developed countries. As China’s population is experiencing an accelerating aging process, how to promote the transformation of the demographic bonus to a health bonus and effectively formulate a deferred-retirement policy has become an urgent problem in China. This paper examines factors influencing older people’s willingness to delay retirement from the perspective of social media. The results of the study provide relevant references for solving this problem.

Our study shows that for older workers, the willingness to delay retirement is mainly affected by work ability and external work stress. Social support gained from social media can effectively help older people enhance their work ability and ease work stress. The results of the model hypothesis test provide the characteristics of older workers’ need of social support.

Based on the findings of this paper, we suggest that the government create deferred-retirement plans based on different occupations and demographic characteristics. We have put forward proposals to extend people’s working lives and help governments implement health bonus policies. Older adults themselves can also actively use social media to improve their social support and physical and psychological health.

## Appendix

Multimedia Appendix 1 Measurement scale.

Multimedia Appendix 2 Indirect and total effects of social media at work on willingness to delay retirement.

## Abbreviations

AVE: average variance extracted
PLS-SEM: partial least squares structural equation modeling
