# Supplementary material for: How Social Media Use at Work Affects Improvement of Older People’s Willingness to Delay Retirement During Transfer From Demographic Bonus to Health Bonus: Causal Relationship Empirical Study
Source: J Med Internet Res. 2021 Feb 10;23(2):e18264. doi: 10.2196/18264 (PMC7904398; doi:10.2196/18264)
Supplement: Multimedia Appendix 1 [file jmir_v23i2e18264_app1.docx]

Appendix 1: Measurement scale

| Construct | Items |  | **Reference** |
| --- | --- | --- | --- |
| Social Media at Work  (SMW) | SMW1 | What is your frequency of usage of social media in the workplace? Not at all (1) -Frequently (7) | Tulu (2017) [52]  Leftheriotis and Giannakos (2014) [9]  Demircioglu and Chen (2019) [53] |
|  | SMW2 | Using social media at work can improve my work efficiency |  |
|  | SMW3 | Using social media at work can improve my communication skills |  |
|  | SMW4 | Using social media at work can help me relieve work stress |  |
|  | SMW5 | I will use social media for entertainment at work |  |
|  | SMW6 | I often use social media to obtain work-related information and knowledge |  |
| Work Ability Index  (WAI) | WAI1 | Suppose you are rated at 7 points when you have the best ability to work. How would you rate your current ability to work? | Martus et al (2010) [54]  Li et al (2015) [55] |
|  | WAI2 | I am very satisfied with my current performance at work |  |
|  | WAI3 | I think my current brain power adapts to the current work requirements |  |
|  | WAI4 | I think my current physical strength adapts to the current work requirements |  |
| Willingness to Delay Retirement  (WDR) | WDR1 | I am happy to keep working |  |
|  | WDR2 | I am confident I will maintain my current working status in the future |  |
|  | WDR3 | I hope to retire now (R) |  |
| Emotional Support  (ES) | ES1 | There is someone (colleagues or leaders) I can talk to about the pressures in my work | Hobman et al (2009) [57]  Ju et al (2015) [58] |
|  | ES2 | I can trust my colleagues or leaders |  |
|  | ES3 | When I have difficulties, some people (leaders or colleagues) are a real source of comfort for me |  |
|  | ES4 | Some people (leaders or colleagues) care about me in my work |  |
| Information Support  (IS) | IS | My colleagues or leaders are able to provide me with the information I need to complete the work |  |
|  | IS | My colleagues or leaders will share with me new knowledge or skills |  |
|  | IS | When I encounter problems, some people (leaders or colleagues) will give me knowledge or information help |  |
|  | IS | There being no one to help me, I have to do it all alone at work (R) |  |
| Work Stress  (WS) | WS1 | My work requires working very hard | Cheng, Luh, and Guo (2003) [67] |
|  | WS2 | My work requires me to work very fast |  |
|  | WS3 | I have to work overtime often |  |
|  | WS4 | I am not asked to do an excessive amount of work (R) |  |
| Self-efficacy  (SE) | SE1 | If I try my best, I can always solve the problem | Cheung and Sun (1999) [61] |
|  | SE2 | It’s easy for me to stick to my ideals and achieve my goals |  |
|  | SE3 | I am confident that I can effectively deal with any sudden events |  |
|  | SE4 | If I put in the necessary efforts, I will be able to solve most of the problems |  |
