# Supplementary material for: How Social Media Use at Work Affects Improvement of Older People’s Willingness to Delay Retirement During Transfer From Demographic Bonus to Health Bonus: Causal Relationship Empirical Study
Source: J Med Internet Res. 2021 Feb 10;23(2):e18264. doi: 10.2196/18264 (PMC7904398; doi:10.2196/18264)
Supplement: Multimedia Appendix 2 [file jmir_v23i2e18264_app2.docx]

Appendix 2: Indirect and total effects of social media at work on willingness to delay retirement

| Path | Standardized path coefficients | T value | p value |
| --- | --- | --- | --- |
| Indirect effect |  |  |  |
| SMW -> IS -> WDR | 0.056 | 4.115 | <0.001 |
| SMW -> IS -> WS -> WDR | 0.038 | 4.698 | <0.001 |
| SMW -> IS -> WAI -> 未WDR | 0.027 | 3.858 | <0.001 |
| SMW -> IS -> SE -> WS -> WDR | 0.021 | 4.587 | <0.001 |
| SMW -> IS -> SE -> WAI -> WDR | 0.02 | 4.838 | <0.001 |
| SMW -> ES -> WDR | 0.02 | 2.018 | 0.044 |
| SMW -> ES -> WS -> WDR | 0.041 | 5.605 | <0.001 |
| SMW -> ES -> WAI -> WDR | 0.039 | 5.342 | <0.001 |
| SMW -> ES -> SE -> WS -> WDR | 0.002 | 1.8 | 0.072 |
| SMW -> ES -> SE -> WAI -> WDR | 0.002 | 1.81 | 0.07 |
| SMW -> SE -> WS -> WDR | 0.016 | 3.753 | <0.001 |
| SMW -> SE -> WAI -> WDR | 0.016 | 3.796 | <0.001 |
| Total effect |  |  |  |
| SMW -> WDR | 0.299 | 10.537 | <0.001 |

*Note: Work ability index = WAI; work stress = WS; self-efficacy = SE; willingness to delay retirement = WDR; information support = IS; emotional support = ES; social media at work = SMW.*
